# Upper tract urothelial carcinoma in crossed fused renal ectopia: Case report and review of the literature

**DOI:** 10.1016/j.eucr.2025.103170

**Published:** 2025-08-19

**Authors:** Xiaoxuan Bai, Suzhan Lv, Dan Liu, Yuexin Liu, Hao Ping

**Affiliations:** Department of Urology, Beijing Tongren Hospital, Capital Medical University, Beijing, 100730, China

**Keywords:** Crossed fused renal ectopia, Upper tract urothelial carcinoma, Radical laparoscopic nephroureterectomy

## Abstract

Upper tract urothelial carcinoma in conjunction with congenital renal malformations is exceedingly rare in clinical practice, presenting significant challenges for surgical intervention. We detail a case involving crossed fused renal ectopia accompanied by upper tract urothelial carcinoma. Computed tomography urography imaging revealed abnormalities in the ureteropelvic region. Subsequent urinary cytology, ureteroscopy and pathological analysis confirmed the presence of urothelial carcinoma. A radical laparoscopic nephroureterectomy was conducted, and postoperative pathology indicated a low-grade urothelial carcinoma. No tumor recurrence or metastasis was observed during one-year postoperative follow-up. Our clinical validation confirms laparoscopic surgery as a safe and efficacious therapeutic modality.

## 1 Introduction

Crossed fused renal ectopia is an uncommon congenital anomaly characterized by the fusion of kidneys, where one kidney translocates across the midline from its original position to the opposite side, merging with the contralateral kidney, while the ureteral orifice in the bladder remains on its original side.^1^ Very little is known about embryogenesis, treatment and prognosis of crossed fused renal ectopia. In the majority of instances, crossed fused renal ectopia remains asymptomatic and is often incidentally discovered during imaging examinations. Some cases may manifest with symptoms such as calculi and infection. However, upper tract urothelial carcinoma in such cases is extremely rare, with few reports available. The combination of crossed fused renal ectopia and urothelial carcinoma complicates preoperative assessment and increases surgical risks due to altered anatomy. This report presents a case of upper tract urothelial carcinoma in right-to-left crossed fused renal ectopia and the first laparoscopic nephroureterectomy for this cancer, highlighting the need for customized preoperative planning and the benefits of minimally invasive methods in preserving kidney function and reducing perioperative risks.

## 2 Materials and methods

**Fig. 1:**
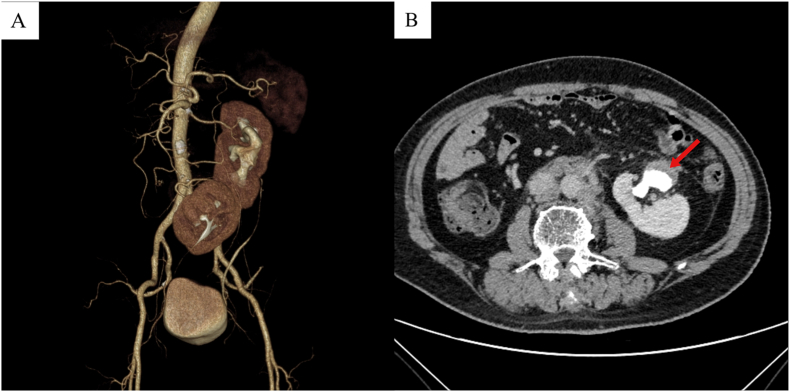
3D CT urography (CTU) imaging demonstrates the right kidney has crossed the midline, becoming inferiorly displaced and fused with the left kidney (A). Excretory phase CTU reveals a filling defect and a soft tissue density shadow in the left pelvis (B).

A 73-year-old male was admitted to the emergency department of our clinic on April 7, 2024, presenting with a one-year history of intermittent gross hematuria and left lower abdominal pain for the past ten days. The physical examination did not reveal any significant positive findings. An abdominal computed tomography urography scan revealed the presence of small calculi in the left ureter, accompanied by left hydronephrosis. Notably, the morphology of both kidneys appeared abnormal, raising a significant suspicion of a fused kidney with malrotation (Fig. 1). Furthermore, a filling defect and a soft tissue density shadow (approximately 2.9 × 2 × 1.5 cm) were observed in the transitional zone of the left renal pelvis and ureter (upper pole of the fused kidney). These findings are indicative of a potentially malignant space-occupying lesion in the left renal pelvis. Urinary cytology confirmed the presence of tumor cells. Ureteroscopic examination identified multiple cauliflower-like masses in the transitional area of the left renal pelvis and ureter, and subsequent biopsy confirmed the diagnosis of low-grade invasive urothelial carcinoma. A computed tomography examination of the chest did not demonstrate any evident pulmonary metastasis. The glomerular filtration rates for the left and right kidneys were 51.21 ml/min and 30.89 ml/min, respectively.

Subsequently, a laparoscopic left nephroureterectomy was performed under general anesthesia. The patient was placed in the right lateral decubitus position. Laparoscopic port placement was planned to accommodate the unusual anatomy and to facilitate adequate exposure. A total of five trocars were used: a 5-mm trocar was inserted at the intersection of the rectus abdominis and the costal margin; another 5-mm trocar was placed below the costal margin along the anterior axillary line; a 12-mm trocar was positioned at the reverse McBurney's point; a 10-mm trocar (camera port) was placed at or adjacent to the umbilicus; and a 12-mm trocar was positioned midway between the umbilicus and the pubic symphysis. This arrangement provided optimal triangulation for mobilization of the fused renal hilum, isthmus, distal ureter, and bladder cuff. During the intraoperative procedure, the left renal hilum was accessed, allowing for the visualization of the left renal artery and vein. Subsequent to the ligation and transection of these vessels, dissection advanced inferiorly to facilitate the mobilization of the left ureter, which was subsequently ligated using a Hem-o-lok clip. The dissection extended into the pelvic region, where the renal isthmus of the fused kidney was identified. The arterial supply located posterior to the isthmus was ligated and divided. Complete mobilization of the left kidney was achieved. The renal isthmus was sutured and subsequently divided to minimize hemorrhage. For the management of the distal ureter and bladder cuff in the setting of altered anatomy due to crossed fused renal ectopia, the distal ureter was meticulously dissected down to the ureterovesical junction. The bladder cuff was excised in an en bloc fashion using a combination of cold scissors and ultrasonic scalpel to ensure complete removal of the intramural ureter. The bladder defect was then closed in two layers with interrupted 3–0 absorbable sutures to achieve a watertight closure and minimize the risk of postoperative leakage. The entire left kidney, ureter and bladder cuff were removed en bloc and submitted for pathological evaluation.

## 3 Results

**Fig. 2:**
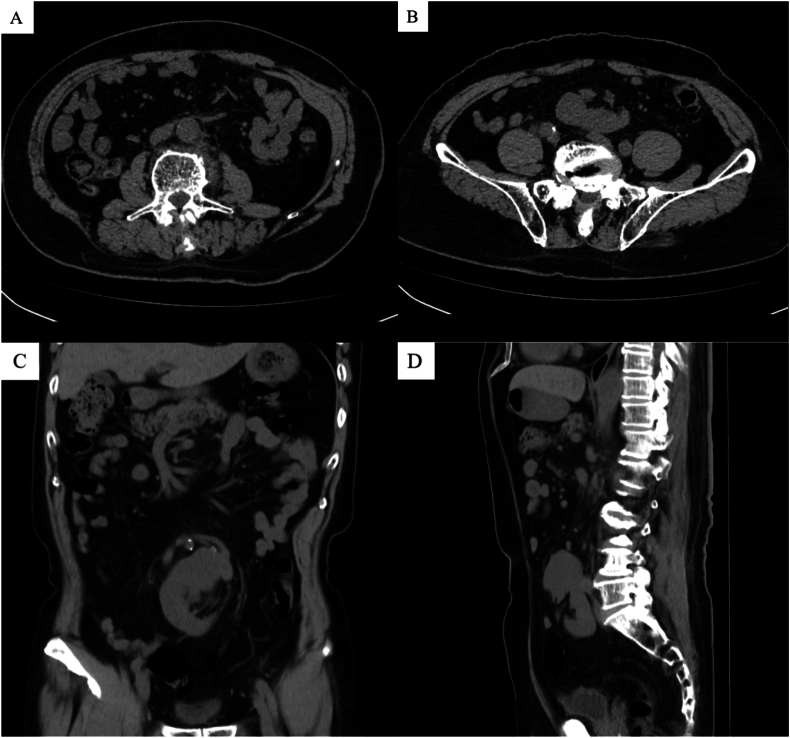
No tumor recurrence or metastasis was observed during the 1-year follow-up. (A, B: transverse plane, C: coronal plane, D: sagittal plane).

The procedure was successful. The estimated intraoperative blood loss was approximately 200 ml, and the surgery duration was approximately 3 hours. Postoperative pathological analysis identified a cauliflower-like tumor protruding from the renal pelvis, measuring approximately 2.5 × 2 × 1.5 cm. Microscopic analysis corroborated the diagnosis of low-grade urothelial carcinoma, with no evidence of invasion into the muscularis propria. The surgical margins were clear of tumor involvement. Preoperative serum creatinine levels were within normal range, whereas postoperative levels increased to 158 μmol/L on the third day, and subsequently remained at around 160 μmol/L. No tumor recurrence or metastasis was observed during the 1-year follow-up (Fig. 2).

## 4 Discussion

Crossed fused renal ectopia is a rare congenital condition where kidneys are fused and misplaced across the midline, complicating diagnosis and treatment, especially with malignancies. In 1957, McDonald et al. categorized crossed fused renal ectopia into six distinct subtypes: unilateral fused kidney (inferior ectopia), sigmoid or S-shaped kidney, lump kidney, L-shaped kidney, disc kidney and unilateral fused kidney (superior ectopia).^2^ The reported incidence of crossed fused renal ectopia varies across different studies, ranging from approximately 1 in 1000 to 7500 cases,^3^^,^^4^ which is significantly lower than the incidence of horseshoe kidney. Crossed fused renal ectopia can be categorized based on the direction of renal displacement into left-to-right and right-to-left types, with an approximate ratio of 3:1.^1^ Furthermore, the condition exhibits a higher prevalence in males compared to females, with a male-to-female ratio of approximately 2:1^5^^.^ The pathogenesis of Crossed fused renal ectopia remains largely undetermined. Two commonly accepted theories are the mechanical theory and the ureteral theory. According to mechanical theory, aberrant umbilical arterial morphogenesis may generate compressive forces that impede cephalad renal migration, thereby redirecting organogenesis toward the contralateral hemipelvis with reduced mechanical resistance. According to ureteral theory, excessive ureteric curvature coupled with disrupted embryonic caudal rotation prevents ipsilateral metanephric blastema-ureteric bud integration, consequently inducing paradoxical contralateral developmental orientation.

There are limited reports concerning Crossed fused renal ectopia associated with tumors. In 2016, Rac G et al. documented a case involving a crossed ectopic fusion kidney with nephroblastoma, which was managed through open partial nephrectomy.^6^ Our review identified four analogous cases: two instances of ureteral cancer,^7^^,^^8^ one instance of renal pelvis cancer,^9^ and one instance of ureteral cancer concomitant with bladder cancer^10^ (Table 1). In three of these cases, surgeons elected to perform open surgical procedures. Drawing upon the preceding literature reviews, this study presents a case of upper tract urothelial carcinoma occurring in the right to left crossed fused renal ectopia. In contrast to the initial case, the current treatment approach utilized laparoscopic surgery.

**Table 1:** 4 cases of crossed fused renal ectopia with urothelial carcinoma.

No	Authors	Published year	Sex	Age	Classification of UC	Classification of CFRE	Surgical approach	grade	stage
1	Gur U.	2003	male	71	ureteral carcinoma	left-to-right	open	high	T1
2	Simforoosh N.	2011	male	74	ureteral carcinoma	left-to-right	laparoscope	low	T1
3	Hearn J.	2020	male	64	renal pelvic carcinoma	right-to-left	open	high	T3
4	Greenberg D.	2024	male	65	ureteral carcinoma and bladder cancer	left-to-right	open	high	T1

UC: urothelial carcinoma, CFRE: crossed fused renal ectopia.

The atypical and intricate anatomical configuration of the kidney renders surgical intervention particularly hazardous and challenging. The vascular architecture of a fused kidney typically presents significant complexity, posing a challenge for surgeons to accurately identify the arteries and veins associated with the affected kidney during surgical procedures. Additionally, the dissection of the isthmus in a fused kidney represents another operative challenge. The arterial supply and venous drainage of the renal isthmus exhibit considerable variability, necessitating a comprehensive assessment integrating vascular anatomy, degree of perivascular adhesions, isthmus hypertrophy, and operator experience to stratify intraoperative hemorrhage risk, thereby enabling preemptive contingency planning. During isthmic dissection, meticulous circumferential mobilization of fibrovascular bundles must precede any transection maneuvers, with definitive vascular control verification being mandatory prior to division. Notably, elevated isthmic tension in select cases may predispose to parenchymal laceration and vascular avulsion during transection, underscoring the imperative for pre-deployment of advanced hemostatic strategies including but not limited to reinforced suture ligation techniques.

Kidney-sparing surgery, including endoscopic ablation, is recommended in low-risk UTUC, defined as unifocal tumors <2 cm, low-grade histology, and absence of invasive features. In our case, although histology confirmed a low-grade tumor, the lesion measured approximately 2.9 cm and involved both the renal pelvis and the ureteral transitional zone, exceeding the recommended size for endoscopic ablation. Moreover, the complex vascular and collecting system anatomy of crossed fused renal ectopia posed a higher risk of incomplete resection and recurrence. Therefore, laparoscopic radical nephroureterectomy was considered more appropriate to achieve oncological control.

Based on the risk stratification, the patient was classified as high-risk UTUC due to tumor size >2 cm despite low-grade histology. Preoperative imaging revealed no suspicious lymphadenopathy, and intraoperative exploration found no grossly enlarged lymph nodes; therefore, lymph node dissection was not performed. However, we acknowledge that lymphadenectomy may be considered in high-risk cases, particularly if there is radiologic or intraoperative suspicion of nodal involvement.

## 5 Conclusions

In this report, we present a case of crossed fused renal ectopia complicated by upper tract urothelial carcinoma. We conclude that upper tract urothelial carcinoma in crossed fused renal ectopia is an extremely rare and unknown disease and laparoscopic surgery is a surgical option because of its low blood loss and complications. We strongly advocate for comprehensive diagnostic evaluations in patients with suspected structural abnormalities of the urinary system to mitigate risks of misdiagnosis or diagnostic oversight. Meticulous preoperative strategizing demonstrates paramount significance in ensuring procedural efficacy and optimizing long-term patient outcomes. Given the rare occurrence of this condition and the elusive pathogenesis underlying its development, concerted efforts in both basic scientific investigations and multicenter clinical trials are imperative.

## CRediT authorship contribution statement

**Xiaoxuan Bai:** Writing – original draft, Investigation, Data curation, Conceptualization. **Suzhan Lv:** Writing – original draft, Investigation, Data curation. **Dan Liu:** Investigation. **Yuexin Liu:** Supervision. **Hao Ping:** Writing – review & editing, Supervision, Funding acquisition.

## Statement of ethics

Clinical trial number: not applicable. Written informed consent was obtained from the patient for publication of this case report and any accompanying images.

## Data availability statement

Data are available on request to the authors.

## Funding sources

This study was funded by 10.13039/501100002855Ministry of Science and Technology of the People's Republic of China (Grant No. 2023YFC2507000).

## Conflict of interest statement

The authors have no conflicts of interest to declare.
